# An Integrated Intelligent Approach for Monitoring and Management of a Deep Foundation Pit in a Subway Station

**DOI:** 10.3390/s22228737

**Published:** 2022-11-11

**Authors:** Chengyu Hong, Jinyang Zhang, Weibin Chen

**Affiliations:** 1 College of Civil and Transportation Engineering, Shenzhen University, Shenzhen 518060, China; 2 Underground Polis of Academy, Shenzhen University, Shenzhen 518060, China; 3 Shenzhen Key Laboratory of Green, Efficient and Intelligent Construction of Underground Metro Station, Shenzhen 518060, China; 4 Key Laboratory for Resilient Infrastructures of Coastal Cities (MOE), Shenzhen University, Shenzhen 518060, China

**Keywords:** intelligent foundation pit, transparent geology, internet of things, machine learning, digital twin

## Abstract

As the scale of foundation pit projects of subway stations in Shenzhen becomes larger, and the construction constraints become more and more complex, there is an urgent need for intelligent monitoring and safety management of foundation pits. In this study, an integrated intelligent approach for monitoring and management of a deep foundation pit in a subway station was proposed and a case study based on the Waterlands Resort East Station Project of Shenzhen Metro Line 12 was used for validation. The present study first proposed the path of intelligent foundation pit engineering. Based on geotechnical survey and building information modeling, a three-dimensional transparent geological model of foundation pit was constructed. Multi-source sensing technologies were integrated, including micro electromechanical system sensing technology, Brillouin optical frequency domain analysis sensing technology, an unmanned aerial vehicle and machine vision for real-time high-precision wireless monitoring of the foundation pit. Moreover, machine learning models were developed for predicting key parameters of foundation pits. Finally, a digital twin integrated platform was developed for the management of the subway foundation pit in both construction and maintenance phases. This typical case study is expected to improve the construction, maintenance and management level of foundation pits in subway stations.

## 1. Introduction

In recent decades, various advanced technologies, such as artificial intelligence (AI), internet of things (IoTs), digital twin (DT), and machine learning (ML), have been successfully applied in various activities of civil engineering, such as slope stability analysis [1,2], geotechnical centrifuges [3], the geological survey industry [4], acquisition and prediction of soil and geotechnical structure properties [5,6,7,8,9,10,11,12], road construction [13], structural health monitoring [14,15], civil infrastructure systems [16] and smart city design [17,18,19]. 

Foundation pit engineering, as one of civil engineering branches, needs to adapt to increasingly complex urban environmental conditions [20] and strict deformation requirements [21], especially in Shenzhen, China [22]. It is urgent to develop multi domain safety management of the whole life cycle of foundation pit engineering. Various intelligent monitoring systems [23,24,25,26] have been developed to improve the effect of foundation pit engineering safety management. In addition, with the development of intelligent foundation pit engineering, advanced technologies have been widely used in the projects, such as transparent geology [27] and multi-source perception [28]. At the same time, ML algorithms, including support vector machines [29,30,31], an artificial bee colony back-propagation model [32,33,34], particle swarm optimization [31,35,36], random forest [37,38], and a hybrid neural network [39,40,41], have been used to predict key parameters of the deep foundation pit. Based on the algorithms, DT was applied to projects on deep foundation pits of metro stations, during the period of construction [42] and management [43].

**Figure 1:**
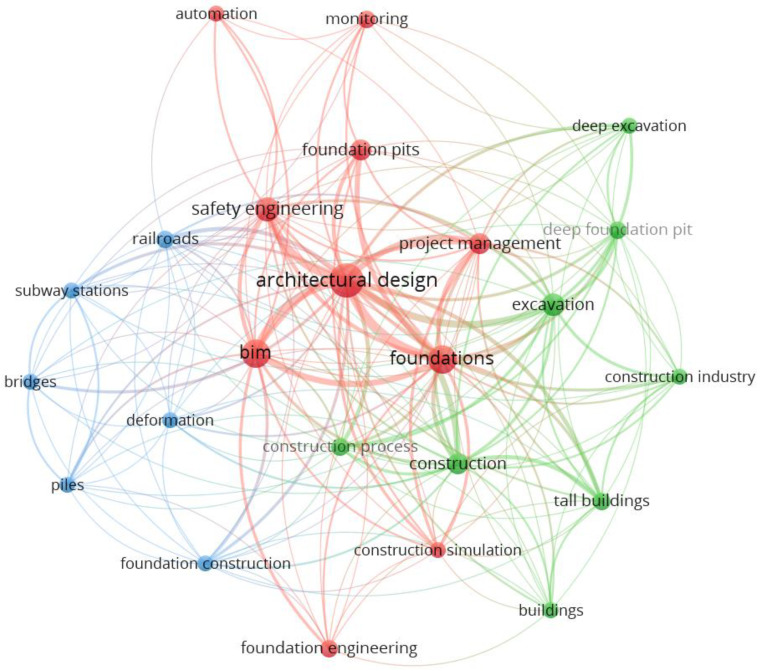
Mapping analysis of co-occurrence of keywords related to intelligent foundation pit engineering.

In this study, 35 published papers related to foundation pit engineering were collected for knowledge mapping. The input keywords in the Scopus search engine included foundation engineering, foundation pit, foundation excavation, foundation construction, BIM, monitoring and automation. A knowledge map of the relationship between the keywords among those literatures was obtained using VOSviewer, as shown in Figure 1. According to the occurrence of keywords, the network as composed of three clusters (color areas), 24 items (keywords) and 51 links. The curve width connecting two items is proportional to the proximity between them. It was found that current technology of foundation pit engineering is closely related to BIM, whereas the technology of monitoring and automation are seldom applied to foundation pit engineering.

Based on the keyword relevance analysis atlas of intelligent foundation pit engineering, and the analysis of previous engineering cases, it was found that the research content of intelligent foundation pit engineering is often limited to a certain part of the project. For example, through the establishment of computer in-situ 3D transparent geology of the foundation pit, soil information can be retrieved and managed with high accuracy [44], and the management of the soil of foundation pit engineering can be accomplished [45], which is convenient for assessing the safety risk of foundation pit engineering [46,47,48]. At present, there has been very little study reported on the intelligent monitoring and management scheme for the whole life cycle of foundation pit engineering.

This study was based on the project of Waterlands Resort East Station of Metro Line 12, Shenzhen, China. The first intelligent foundation pit integrating “transparent geology multi-source perception deep learning digital twin” was done through in-situ testing, BIM, multi-source perceptions, ML, and DT. This innovation can significantly enhance the design, operation, and management of foundation pit projects for metro stations.

## 2. Implementation Path of Intelligent Foundation Pit Engineering

**Figure 2:**
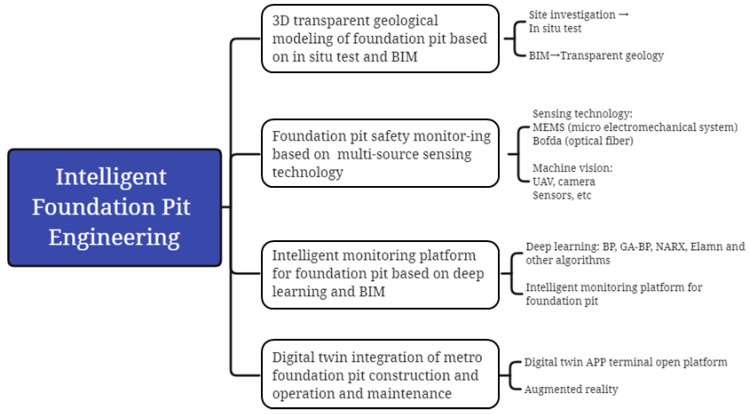
Implementation path of intelligent foundation pit engineering.

Figure 2 shows the implementation path of intelligent foundation pit engineering. First, through geo-technical engineering investigation (especially in-situ test), the site engineering geological information was obtained and the application programming interface (API) of BIM software (Revit2020, Autodesk, Inc., San Rafael, CA, USA) was used to create a three-dimensional geometric structure of the soil layer and interface. Transparent geological retrieval was used to manage the soil layer information. Second, multi-source sensing technologies, such as a micro electro mechanical system (MEMS) [49], Brillouin optical frequency domain analysis (BOFDA) distributed optical fiber [50], an unmanned aerial vehicle (UAV) [51] and machine vision (MV) [52], were carried out to ensure the accuracy and reliability of field monitoring data. Furthermore, four kinds of neural network algorithms were used to predict the field working condition parameters and compared with real data. Finally, an intelligent monitoring platform of foundation pit engineering based on the independent intellectual property BIM was used to monitor and predict the change trend of key parameters of foundation pit engineering. At the same time, a DT application terminal open platform integrating intelligent construction and maintenance of rail transit was used for the monitoring, prediction and management of the whole life cycle of foundation pit engineering. Augmented reality technology was used to conduct on-site visual management.

## 3. Project Overview

**Figure 3:**
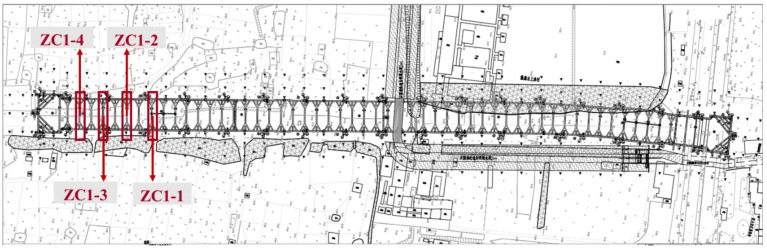
Waterlands Resort East Station Project of Shenzhen Metro Line 12.

This study was based on the project of Waterlands Resort East Station of Metro Line 12, Shenzhen, China. An overview of the project is shown in Figure 3. The station is a transfer station that is underground. It is primarily made up of the station’s main structure (the platform, station hall, production area, and living room), as well as the entrance, exit, ventilation channel, and ground air pavilion. The station’s main structure is 543.7 m long and 19.7 m broad. The common part is 21.1 m wide. The building is 17.5–18.5 m deep and 13.83 m high. The station uses an open excavation approach for construction and a ground-connecting wall support system for the foundation pit. Undeveloped land is currently present in the area surrounding the station. The supports instrumented with distributed optical fibers are sequentially represented by ZC1-1 to ZC1-4.

## 4. Three-Dimensional Transparent Geological Modeling of Foundation Pit Based on In-Situ Test and BIM

**Figure 4:**
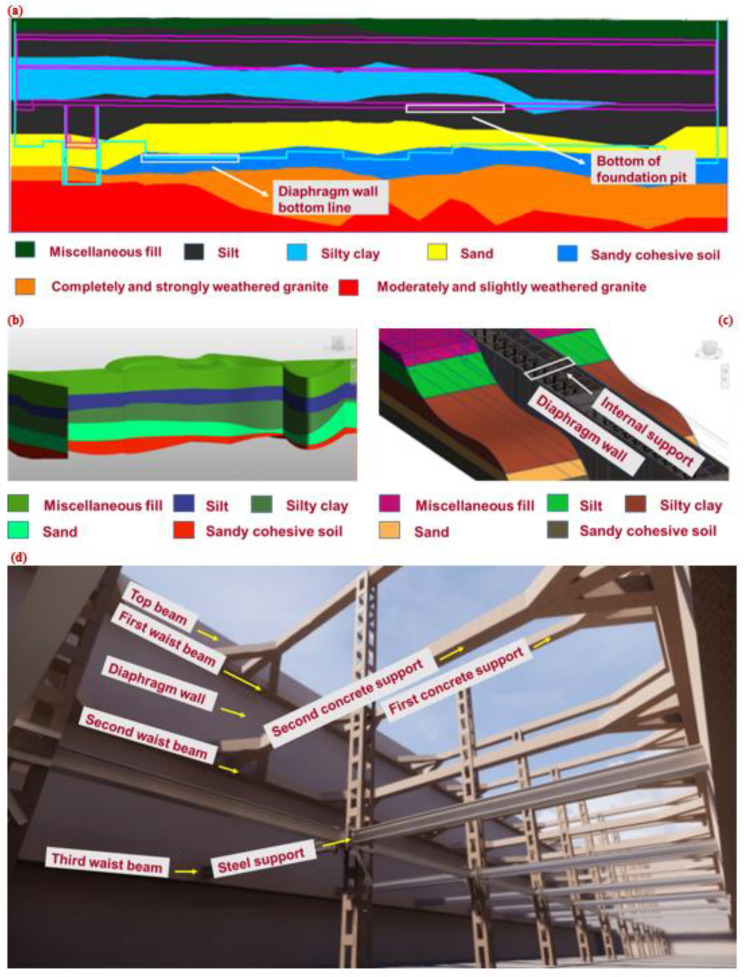
Modeling the diaphragm wall and internal support structure in the soil layer and deep foundation pit. (**a**) site geological longitudinal section; (**b**) in situ 3D transparent geological model; (**c**) model of diaphragm wall and internal support structure in deep foundation pit; (**d**) final rendering effect of foundation pit.

Through a geotechnical engineering investigation (especially in-situ test), on-site engineering geological basic information data was obtained. The three-dimensional geological information and foundation pit engineering structure in the current project were modeled with BIM technology to form a three-dimensional transparent foundation pit engineering infrastructure. Based on the deep foundation pit of Waterlands Resort East Station Project of Shenzhen Metro Line 12, this study carried out six in-situ tests, including a double bridge static penetration test, a three-bridge static penetration test, a pressure meter test, a flat shovel lateral expansion test, a vane shear test and a full flow penetration test. The change laws of the strength, permeability coefficient and mechanical parameters of the stratum soil mass, and the fine division of the soil layer and relevant geotechnical parameters, were analyzed based on the test results. As shown in Figure 4, the soil stratum, diaphragm wall and internal support structure of deep foundation pit were model using C# programming and the built-in API of BIM software (Revit2020, Autodesk, Inc., San Rafael, CA, USA). Through C# programming combined with the API provided by Autodesk Revit, and completed exploration hole survey data files (.xlsx format), the corresponding point positions of the exploration holes were generated in the conceptual volume and formed by point-to-point connection model lines, which further generated 3D models of soil layers by generating shapes. Figure 4a shows the field geological longitudinal section obtained by the in-situ test, and Figure 4b shows the in-situ three-dimensional transparent geological model. Figure 4c shows a model of the diaphragm wall and internal support structure of the deep foundation pit, while Figure 4d illustrates the final rendering effect of the foundation pit. As shown in Figure 4, it is worth noting that the modeling of the soil layer has been not intergraded into the analysis of the foundation pit safety in the current project. Despite this limitation, the successful development and application of three-dimensional transparent foundation pit engineering will improve the effect of engineering safety management and facilitate decision-making of the intelligent construction.

## 5. Foundation Pit Safety Monitoring Based on Multi-Source Sensing Technology

Through multi-source sensing technologies, including MEMS, BOFDA distributed optical fiber, UAV and MV, a safety monitoring assessment of foundation pit engineering was carried out. 

### 5.1. Foundation Pit Monitoring Based on MEMS Sensing Technology

**Figure 5:**
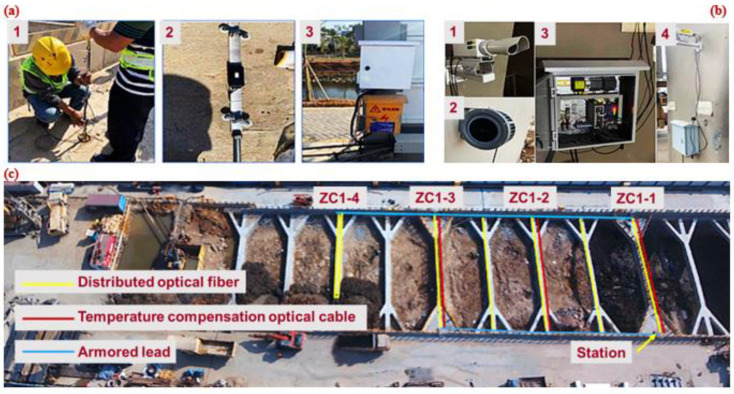
(**a**) Field installation of MEMS inclinometer sensor. (1) Layout sensor. (2) Lowering the MEMS sensor vertically. (3) Station protection. (**b**) On-site installation of machine vision sensor system: (1) camera; (2) target; (3) power supply and LTE communication module; (4) machine vision sensor. (**c**) UAV monitoring on-site working conditions.

**Figure 6:**
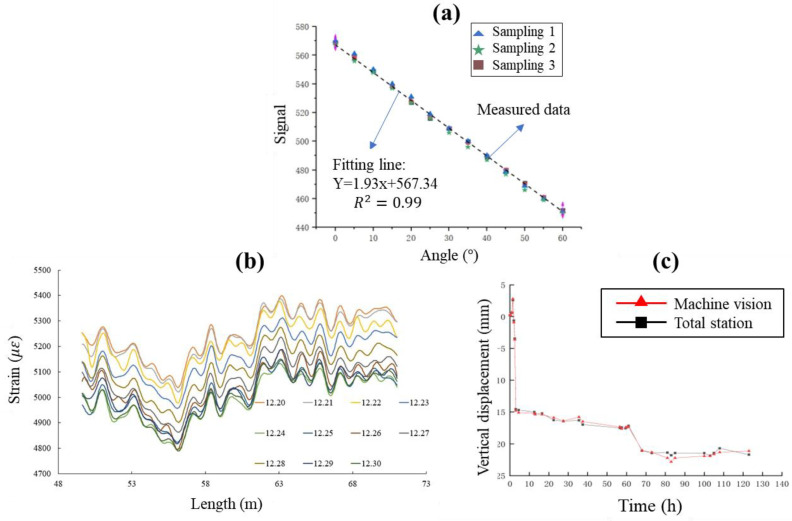
(**a**) Typical calibration results for MEMS technology. (**b**) Representative BOFDA results for ZC1-2. (**c**) Measurement comparison between machine vision and total station.

MEMS technology is mainly characterized by miniaturization and integration [49]. Sensors developed based on MEMS technology are characterized by high accuracy and low power consumption [53,54]. In this project, a wireless inclination sensor based on MEMS technology was used to monitor the horizontal displacement of the foundation pit support. As shown in Figure 5a, MEMS inclination sensors, including monitoring and sensing equipment and automatic data acquisition instrument, were installed at the construction site of the deep foundation pit. Typical calibration results are shown in Figure 6a. As shown in Figure 6a, the angle change had a good correlation with the signal change ($R^{2}$ = 0.99), which indicates that it was sufficient to complete the measurement of 0–60° in a single sensor. 

### 5.2. Foundation Pit Monitoring Based on BOFDA Distributed Optical Fiber Sensing Technology

Optical fiber sensing technology meets the requirements of high precision, long-distance and long-term measurement. Distributed optical fiber sensor, represented by BOFDA, achieves continuous spatial distributed measurements with accurate measurement results and small errors [50], and is widely applied to engineering practice [55]. As shown in Figure 3, optical fiber supports were arranged along the erection section on the typical sections ZC1-1 to ZC1-4. Two optical cables were implanted inside the continuous wall to measure strain and temperature. The distributed optical fiber inclinometer tube was placed in sections to measure the horizontal displacement of the soil, and grouting was used to seal the hole so that the inclinometer tube and the pile body could deform cooperatively. Representative BOFDA results for ZC1-2 are illustrated in Figure 6b. It is shown in Figure 6b that the effective measurement length of the support was 21.42 m and the spatial resolution was 0.05 m. Since the optical fiber was pre-stretched before laying, the strain of the strained optical cable monitored by the BOFDA equipment was approximately 5100 με after pre-stretching.

### 5.3. Foundation Pit Settlement and Displacement Monitoring Based on UAV and Machine Vision

At present, UAV [51] and MV [52] technologies are gradually being applied to foundation pit engineering. For example, a UAV has been used to patrol the deep foundation pit excavation site and monitor safety risk early warning. MV has mainly been used for structural displacement monitoring [56]. The MV system includes cameras, lenses, computer processing software and markers. The monitoring work is completed through camera calibration, feature extraction and displacement calculation [52]. Figure 5b shows the installation of a MV sensor system, including camera, target, power supply, LTE communication module and MV sensor. The vertical displacement obtained from MV was compared with that measured by the total station, as shown in Figure 6c. The comparison indicates that the MV is also a good technique for the vertical displacement measurement.

Due to the depletion of the sensor itself, the measurement accuracy declines after a period of time, which affects the accuracy of the monitoring results. Different from the traditional single engineering monitoring method, this study uses MEMS sensing technology, BOFDA distributed optical fiber sensing technology, a UAV and MV technology to monitor the working parameters of the foundation pit, including but not limited to axial force, stress, temperature and displacement, to give reasonable play to the advantages of various sensors and make up for the shortcomings of various sensors, and ensure the accuracy and reliability of monitoring parameters.

## 6. Intelligent Monitoring Platform for a Foundation Pit Based on Deep Learning and BIM

Based on the foundation pit project of Waterlands Resort East Station, an intelligent monitoring platform of foundation pit based on deep learning algorithms and BIM was developed.

### 6.1. Neural Network Models

The back propagation (BP) neural network algorithm is composed of three-layer networks of input layer, hidden layer and output layer. Its core idea is to transmit the output error back to the input layer by layer through the hidden layer in some form [57,58]. Based on the BP neural network, the deformation of deep foundation pit can be predicted [59]. A Genetic Algorithm optimized Back Propagation (GA-BP) neural network model uses the GA genetic algorithm of “survival of the fittest and survival of the fittest” to optimize the BP neural network algorithm, so that the optimized BP algorithm can better predict the function output [60]. In recent years, it has been applied to the prediction of foundation pit settlement [61]. The nonlinear autoregressive network with exogenous inputs (NARX) neural network is composed of four parts: input layer, hidden layer and output layer, and input and output delay [62], which has been applied to the prediction of key parameters of a foundation pit in recent years [63]. The Elman neural network is a dynamic recurrent neural network that has the characteristics of internal feedback, storage and time delay [64], and has been applied in foundation pit deformation prediction in recent years [65]. In summary, the BP network has some defects, such as the slow learning convergence speed, unguaranteed convergence to the global minimum point, and an uncertain network structure. Combined with GA, BP-GA can optimize weights and thresholds. ELMAN has a short-term memory function, which can internally feedback, store and use the output information of the passed time. As a result, ELMAN is better than the BP network in terms of computing power and network stability.

These four neural network models were used to train the axial force, settlement and displacement dataset of the support system of the deep foundation pit over the past one year and then develop prediction models. The reliability of the proposed models was assessed using root mean square error. The proposed models are expected to be used for the early warning and safety assessment of deep foundation pits. By calculating the relative average error, we found these four ML models can accurately predict the axial force, settlement and displacement. Furthermore, the GA-BP algorithm has the best prediction performance due to the smallest error.

### 6.2. Intelligent Monitoring Platform of Foundation Pit Based on BIM

**Figure 7:**
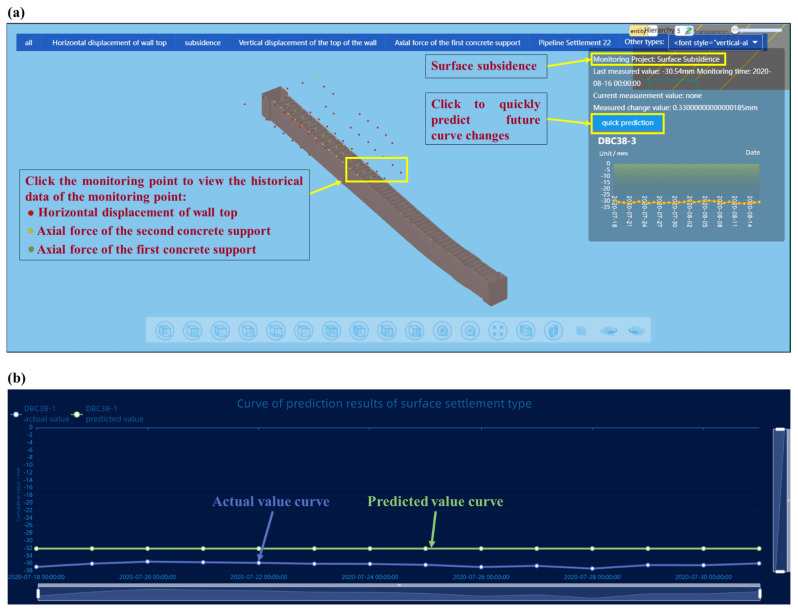
(**a**) Monitoring hotspot map. (**b**) Algorithm back-testing function (comparison of monitoring and prediction curves).

The intelligent monitoring platform of the foundation pit was constructed using IoTs technology and a three-dimensional geographic information system [66]. Figure 7a shows the monitoring hotspot map and historical data of monitoring points in the platform monitoring point data management. The background management of the platform configured the monitoring item information, including the configuration of project monitoring type, the configuration of project basic information and the configuration of project work items. The specific pages include the project overview, construction progress (including project notes and construction progress), construction site monitoring, monitoring management (including monitoring point classification management and data management) and safety prediction.

At present, the platform has only been used to monitor the key parameters during the foundation pit construction stage. It is necessary to complete the monitoring of the whole life cycle of the subway station project using other platforms. Based on the related research of traditional foundation pit engineering monitoring and management, the platform was developed by the Future Underground City Research Institute of Shenzhen University in conjunction with Jinan Bimu Digital Software Technology Co., Ltd. (Jinan, China), which is convenient for enterprise users to monitor and calculate their own projects. Based on the cast modeling platform of Huawei Kunpeng cloud, the platform completes the application and development of BIM graphics and subsequent multi-source heterogeneous data. The parametric model data is uploaded and downloaded through the cast without loss. The platform supports repeated editing and modification of BIM models and can add/delete monitoring equipment models for many times. The real-time synchronization of the platform is conducive to the monitoring and management of the BIM model at different stages, and the reuse of one modeling for many times greatly improves the application value of the model. The platform provides an open cloud computing framework, supports the loading of third-party algorithms, and is used to calculate and analyze the collected data of different monitoring devices online, and predict the future development trend with one click. At the same time, an algorithm back test function is provided to directly compare the predicted value with the measured value. The accuracy of the prediction result is evaluated and the most appropriate algorithm according to the data type is selected. As shown in Figure 7a,b, the platform provides the algorithm back testing function, and selects the prediction time period with the actual monitoring value. Two line graphs are shown in the Figure 7: one line graph for the actual value and one line graph for the predicted value. It is convenient for the user to compare the actual value (blue) and the predicted value (green) and directly assess the reliability of the selected algorithm.

## 7. Digital Twin Integration of Metro Foundation Pit Construction and Operation and Maintenance

Based on Waterlands Resort East Station Project of Shenzhen Metro Line 12, this study developed a DT app terminal open platform integrating intelligent construction and management of rail transit foundation pit. Unity3D was used to realize intelligent upgrading of two-dimensional drawings. The platform accomplished the integration of a station building drawing and model, and all current project drawings of the enterprise could be viewed in the start page. The current platform was divided into two parts: intelligent construction and intelligent management.

**Figure 8:**
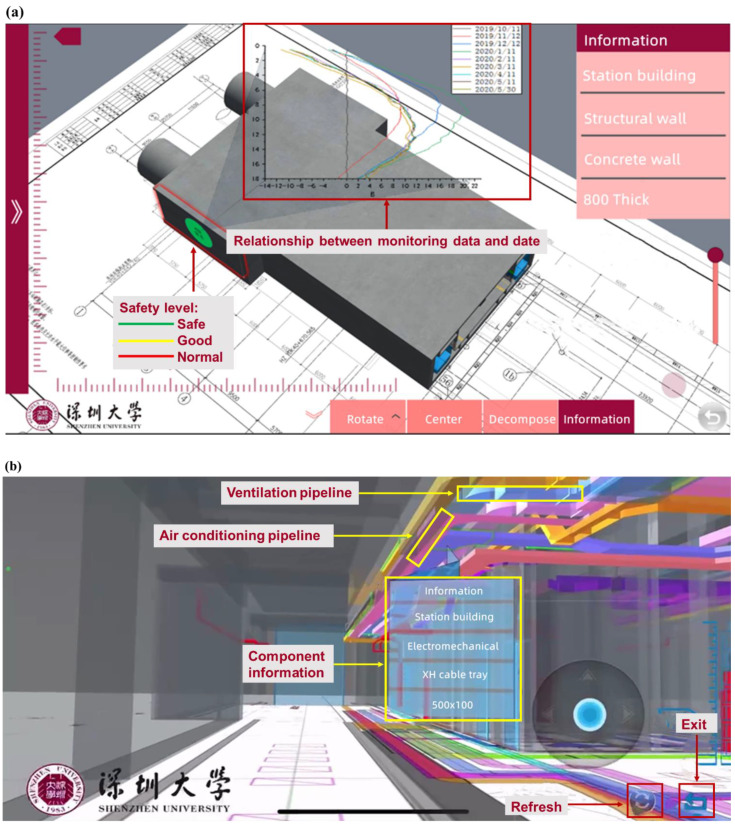
(**a**) Building structure model: holistic model and decomposition model. (**b**) Electromechanical pipeline model, holistic model and decomposition model.

As shown in Figure 8a, the intelligent construction component can access model information and monitoring data. By clicking on the component of the model, we can view detailed information included in the construction, i.e., size, location, name and monitoring data diagram (the past collection data of all monitoring points of the component). In the intelligent management part, we can view the layout of electromechanical equipment in the station building, including the comprehensive plan, ventilation plan, weak current plan, strong current plan and water supply and drainage plan. It is possible to disassemble the station building structure through the model decomposition function, and click the component to view the detailed information of the internal pipeline of the model. As shown in Figure 8b, the roaming function can be used inside the platform app. By clicking on the components in the virtual space, an information bar pops up that includes basic information such as project, engineering, system and size. Here, the construction system information is displayed, and the air conditioning and ventilation pipelines are displayed at the top. Frontline personnel can communicate design requirements more easily and efficiently by roaming in a mixed reality scene containing 2D drawings and 3D information models based on mobile phone viewports.

This app is currently applied only to the integrated intelligent construction and management project of Waterlands Resort East Station Project of Shenzhen Metro Line 12. If it is to be applied to other projects, it needs to import relevant information. At the same time, the compatibility problem of the app still needs to be improved. Currently, it can only be used for mobile phones equipped with the Android system. Different from the traditional DT platform, the app includes three major functions: three-dimensional visualization, full life cycle monitoring and predictive analysis. Three-dimensional visualization breaks the traditional mode of integrating building information through plane drawings, and maps the building model of the physical real world through 3D modeling technology. The information integration of DT from planning and design to construction to operation and maintenance stage ensures the integrity and consistency of data and runs through the whole life cycle of the subway station. The introduction of DT technology provides a basis for predictive decision-making and analysis. The key parameters are monitored by the sensors, and the data collected by the monitoring sensors are analyzed with the help of the deep learning algorithm, and the safety level is predicted at the same time.

**Figure 9:**
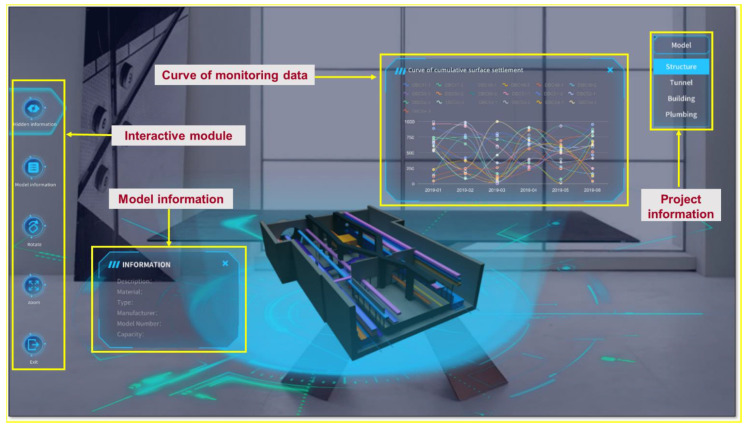
Waterlands Resort East Station Project of Shenzhen Metro Line 12 based on Augmented Reality Technology.

**Figure 10:**
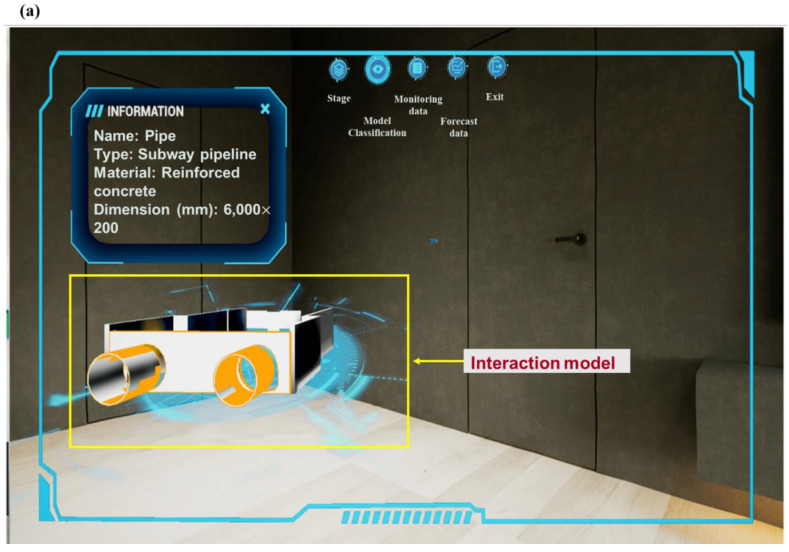

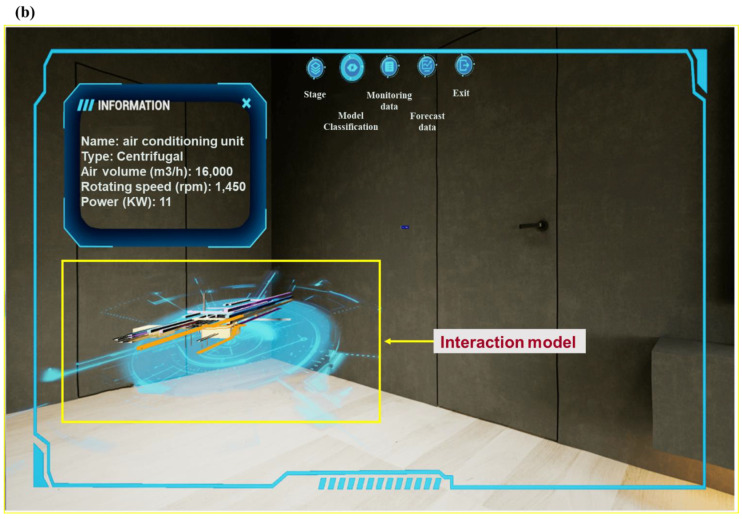
Building structure model and data: (**a**) electromechanical pipeline model and (**b**) air conditioning unit information.

**Figure 11:**
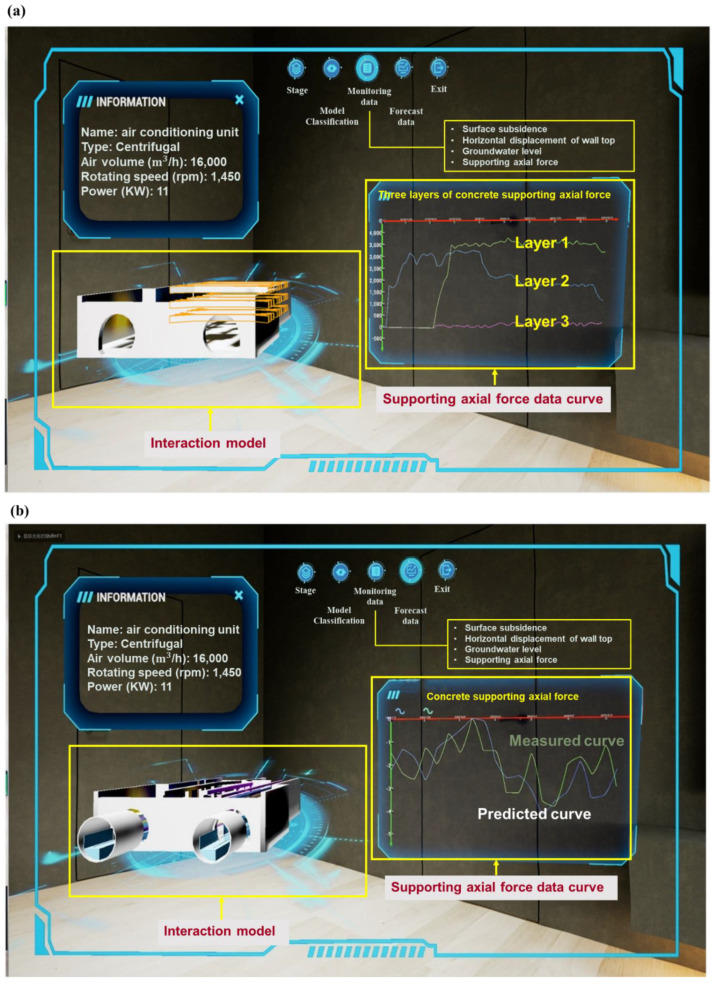
(**a**) Monitoring data of supporting axial force. (**b**) Predicted data of support axial force monitoring point.

Augmented reality combines real world information and virtual world information. Through real-time imaging, three-dimensional and other technologies, it projects physical information that is difficult to experience in a certain time and space range of the real world into a real world in the form of virtual information, so as to be directly captured by human senses and achieve a perceptual experience beyond reality. The working principle of AR includes video capture, graphics system, video synthesis, and video output. As shown in Figure 9, based on the open platform of DT app terminal and augmented reality technology, the BIM 3D model, facilities, equipment and other operation and maintenance data were combined with the current real tunnel scenario to realize the DT of Waterlands Resort East Station Project of Shenzhen Metro Line 12. Information such as surface settlement, vertical displacement of the wall top, horizontal displacement of the wall top, horizontal displacement of pipelines, pipeline settlement, underground water level and axial force of the first concrete support, can be viewed. Figure 10a,b shows the building structure model, electromechanical pipeline model and data information, respectively. We can view the building data information and air conditioning unit information through gesture interaction. Figure 11a,b shows the monitoring data and prediction data of the support axis force. Similarly, different parts of the model to view the corresponding information can be selected by clicking the UI through the gesture interaction function.

## 8. Conclusions

This study advances a new idea for an integrated intelligent approach for monitoring and management of a deep foundation pit in a subway station, which is divided into four modules: the implementation path of intelligent foundation pit engineering, field investigation and in-situ test, multi-source perception technology, ML prediction algorithm and DT integration of foundation pit construction and operation and maintenance. This innovative idea was implemented in the foundation pit engineering of the Waterlands Resort East Station Project of Shenzhen Metro Line 12. The main conclusions are as follows:

1. Through on-site geological investigation, refined geological information of the on-site soil layer was obtained. Three-dimensional transparent geology was then developed on the BIM platform to facilitate information management of the on-site soil layer and effectively control the construction risk;
2. Through MEMS sensing technology, BOFDA distributed optical fiber sensing technology, laser radar, UAV and machine vision technology, the key parameters of the foundation pit, including but not limited to axial force, displacement, strain, temperature, etc., were obtained. The accuracy and reliability of monitoring data were greatly improved using a variety of monitoring methods and multi-source sensing technology;
3. An intelligent monitoring platform of foundation pit based on BIM of independent intellectual property rights was developed using a cast modeling cloud platform to realize integrated monitoring and management of the foundation pit during construction. Four ML neural network algorithms were used to predict the key parameters of the foundation pit. It was found that the algorithm with the smallest error in the project was the GA-BP algorithm;
4. A DT app terminal open platform integrating intelligent construction and operation and maintenance of rail transit was developed using DT means, which included three major functions: three-dimensional visualization, full life cycle monitoring and predictive analysis. Based on app and augmented reality technology, BIM 3D model, facilities, equipment and other operation and maintenance data were combined with the current real tunnel scenario to realize the DT of subway stations.

At present, the data obtained from the in-situ test reflect the real parameters of rock and soil to the maximum extent. The stability of different types of sensors is different, resulting in high cost and long time-consuming of the monitoring platform with multi-source information fusion. Different from the traditional foundation pit project management scheme, this research achieved the integration of construction, operation and maintenance and management of intelligent foundation pit project through four modules of “transparent geology multi-source perception deep learning digital twin”, which intuitively displays the soil layer information, monitoring data, prediction results and three-dimensional models of construction and electromechanical, greatly improving the project management efficiency of engineering personnel.

## Data Availability

All data included in this study are available upon request by contact with the corresponding author.

## References

[1] Hou X. (2018). Geotechnical Engineering Slope Monitoring Based on Internet of Things. Int. J. Online Eng..

[2] Abraham M.T., Satyam N., Pradhan B., Alamri A.M. (2020). IoT-based geotechnical monitoring of unstable slopes for landslide early warning in the Darjeeling Himalayas. Sensors.

[3] Srokosz P. (2017). Internet of Things in geotechnical engineering—An example of application. Inżynieria Morska I Geotech..

[4] Ma Y., Guo G. (2020). Intelligent algorithm of geotechnical test data based on Internet of Things. Comput. Commun..

[5] Kumar B.P., Prasad A. IoT based equipment for real-time identification of soil properties. Proceedings of the 2020 7th International Conference on Smart Structures and Systems (ICSSS).

[6] Carri A., Valletta A., Cavalca E., Savi R., Segalini A. (2021). Advantages of IoT-based geotechnical monitoring systems integrating automatic procedures for data acquisition and elaboration. Sensors.

[7] Wu P., Tan D., Lin S., Chen W., Yin J., Malik N., Li A. (2022). Development of a monitoring and warning system based on optical fiber sensing technology for masonry retaining walls and trees. J. Rock Mech. Geotech. Eng..

[8] Zhu H., Garg A., Yu X.B., Zhou H.W. (2022). Editorial for Internet of Things (IoT) and Artificial Intelligence (AI) in geotechnical engineering. J. Rock Mech. Geotech. Eng..

[9] Attache S., Remadna I., Terrissa L.S., Maouche I., Zerhouni N. (2022). IoT Based Prediction of Active and Passive Earth Pressure Coefficients Using Artificial Neural Networks. Proceedings of the International Conference on Networked Systems.

[10] Tang C., Zhou W., He S. Settlement prediction of immersed tunnels by a Bayesian framework. Proceedings of the 25th Annual Conference of HKSTAM 2022 The 17th Jiangsu–Hong Kong Forum on Mechanics and Its Application.

[11] Chen W.-B., Zhou W.-H., Sadowski Ł., Yin Z.-Y. (2021). Metaheuristic model for the interface shear strength between granular soil and structure considering surface morphology. Comput. Geotech..

[12] Cheng Z.-L., Yang S., Zhao L.-S., Tian C., Zhou W.-H. (2021). Multivariate modeling of soil suction response to various rainfall by multi-gene genetic programing. Acta Geotech..

[13] Meža S., Mauko Pranjić A., Vezočnik R., Osmokrović I., Lenart S. (2021). Digital twins and road construction using secondary raw materials. J. Adv. Transp..

[14] Lu R., Rausch C., Bolpagni M., Brilakis I., Haas C.T. (2020). Geometric accuracy of digital twins for structural health monitoring. Structural Integrity and Failure.

[15] Ye C., Butler L., Calka B., Iangurazov M., Lu Q., Gregory A., Girolami M., Middleton C. A digital twin of bridges for structural health monitoring. Proceedings of the 12th International Workshop on Structural Health Monitoring 2019.

[16] Callcut M., Cerceau Agliozzo J.-P., Varga L., McMillan L. (2021). Digital twins in civil infrastructure systems. Sustainability.

[17] Taylor J.E., Bennett G., Mohammadi N. (2021). Engineering Smarter Cities with Smart City Digital Twins.

[18] Zhou Y., Wei X., Peng Y. (2021). The modelling of digital twins technology in the construction process of prefabricated buildings. Adv. Civ. Eng..

[19] Moretti N., Xie X., Merino Garcia J., Chang J., Kumar Parlikad A. (2021). Developing a Federated Data Model for Built Environment Digital Twins. Comput. Civ. Eng..

[20] Jin X. (2021). Research on design and deformation law of soil rock foundation pit adjacent to a subway station. Chin. J. Undergr. Space Eng..

[21] Wang W., Ding W., Yang X., Zheng G., Xu Z. (2020). Foundation Pit Engineering and Underground Engineering—New Technologies of High Efficiency, Energy Saving, Low Environmental Impact and Sustainable Development. China Civ. Eng. J..

[22] Yang Q., Zhao B., Sun F., Zhao T. (2018). Experimental Study on the Mechanism of Water Level Change Caused by Foundation Pit Dewatering in Shenzhen Subway Station in Typical phreatic Stratum. China Railw. Sci..

[23] Zhang Y.F., Cai M.F. (2012). Geotechnical Engineering Intelligent Monitoring and Controlling System and Its Application in Pit Engineering. Appl. Mech. Mater..

[24] Tian W., Meng J., Zhong X.-J., Tan X. (2021). Intelligent early warning system for construction safety of excavations adjacent to existing metro tunnels. Adv. Civ. Eng..

[25] Wang Y. (2022). Engineering Safety Management System Based on Robot Intelligent Monitoring. Adv. Multimed..

[26] Huang Z., Mao C., Guan S., Tang H., Chen G., Liu Z. (2021). Simulation research on the deformation safety monitoring and evaluation algorithm of coastal soft foundation pit based on big data. Soft Comput..

[27] Zhou C., Ma W., Sui W. (2022). Transparent soil model test of a landslide with umbrella-shaped anchors and different slope angles in response to rapid drawdown. Eng. Geol..

[28] Zhou H., Zhao Y., Shen Q., Yang L., Cai H. (2020). Risk assessment and management via multi-source information fusion for undersea tunnel construction. Autom. Constr..

[29] Li X., Liu X., Li C.Z., Hu Z., Shen G.Q., Huang Z. (2019). Foundation pit displacement monitoring and prediction using least squares support vector machines based on multi-point measurement. Struct. Health Monit..

[30] Song Z., Liu S., Jiang M., Yao S. (2022). Research on the Settlement Prediction Model of Foundation Pit Based on the Improved PSO-SVM Model. Sci. Program..

[31] Yang R., Yuan S. (2022). Monitoring and Prediction of Highway Foundation Settlement Based on Particle Swarm Optimization and Support Vector Machine. Math. Probl. Eng..

[32] Feng T., Wang C., Zhang J., Zhou K., Qiao G. (2022). Prediction of stratum deformation during the excavation of a foundation pit in composite formation based on the artificial bee colony–back-propagation model. Eng. Optim..

[33] Feng T., Wang C., Zhang J., Wang B., Jin Y.-F. (2022). An improved artificial bee colony-random forest (IABC-RF) model for predicting the tunnel deformation due to an adjacent foundation pit excavation. Undergr. Space.

[34] Su G., Zhang K., Zhang H., Zhang Y. Deformation prediction of foundation pit using Gaussian process machine learning. Proceedings of the 2009 Asia-Pacific Conference on Computational Intelligence and Industrial Applications (PACIIA).

[35] Zhu J. (2019). Study on deformation law of foundation pit by multifractal detrended fluctuation analysis and extreme learning machine improved by particle swarm optimization. J. Yangtze River Sci. Res. Inst..

[36] Yang Z., Dong X., Xie D., Cao J. Research on Optimal Design of Foundation Pit Anchor Support based on Improved Particle Swarm Optimization. Proceedings of the IOP Conference Series: Earth and Environmental Science.

[37] Zhou Y., Li S., Zhou C., Luo H. (2019). Intelligent approach based on random forest for safety risk prediction of deep foundation pit in subway stations. J. Comput. Civ. Eng..

[38] Zhang P., Jin Y.-F., Yin Z.-Y., Yang Y. (2020). Random forest based artificial intelligent model for predicting failure envelopes of caisson foundations in sand. Appl. Ocean Res..

[39] Deng R., Zhou L. (2022). Optimising the supporting structure of a bridge’s foundation pit based on hybrid neural network. Int. J. Crit. Infrastruct..

[40] Cui C.-Y., Cui W., Liu S.-W., Ma B. (2021). An optimized neural network with a hybrid GA-ResNN training algorithm: Applications in foundation pit. Arab. J. Geosci..

[41] Liu C., Wang Y., Hu X., Han Y., Zhang X., Du L. (2021). Application of GA-BP neural network optimized by Grey Verhulst model around settlement prediction of foundation pit. Geofluids.

[42] Wang H., Sun Y. (2018). Research on green information construction of deep foundation pit of subway station. J. Liaoning Tech. Univ. Nat. Sci..

[43] Kaewunruen S., Peng S., Phil-Ebosie O. (2020). Digital twin aided sustainability and vulnerability audit for subway stations. Sustainability.

[44] Valeria N., Roberta V., Vittoria C., Domenico A., de Silva Filomena F.S. (2019). A new frontier of BIM process: Geotechnical BIM. Proceedings of the XVII European Conference on Soil Mechanics and Geotechnical Engineering.

[45] Zhao Y. (2019). Geotechnical engineering design and visual analysis based on computer 3D geological model—Review of Geotechnical Engineering Investigation and Design. Chin. J. Geotech. Eng..

[46] Zou Y., Kiviniemi A., Jones S.W. (2017). A review of risk management through BIM and BIM-related technologies. Saf. Sci..

[47] He H., He J., Xiao J., Zhou Y., Liu Y., Li C. (2020). 3D geological modeling and engineering properties of shallow superficial deposits: A case study in Beijing, China. Tunn. Undergr. Space Technol..

[48] Fabozzi S., Biancardo S.A., Veropalumbo R., Bilotta E. (2021). I-BIM based approach for geotechnical and numerical modelling of a conventional tunnel excavation. Tunn. Undergr. Space Technol..

[49] Solgaard O., Godil A.A., Howe R.T., Lee L.P., Peter Y.-A., Zappe H. (2014). Optical MEMS: From micromirrors to complex systems. J. Microelectromech. Syst..

[50] Wang X., Shi B., Wei G., Cheng G., Zhang C. (2015). Performance and Characteristics of BOFDA—A New Monitoring Technology for Civil and Geotechnical Engineering. J. Disaster Prev. Mitig. Eng..

[51] Zhou C., Jiang S., Lin X. (2016). Research on safety risk inspection and early warning of deep foundation pit construction based on unmanned aerial vehicle. Constr. Technol..

[52] Ye X., Dong C. (2019). Survey of structure displacement monitoring based on computer vision. China J. Highw. Transp..

[53] Baloch S.K., Jonáš A., Kiraz A., Alaca B.E., Erkey C. (2018). Determination of composition of ethanol-CO_2_ mixtures at high pressures using frequency response of microcantilevers. J. Supercrit. Fluids.

[54] Zhao L., Huang L., Hu Y., Jiang W., Lu D., Li Z., Zhou X., Wang J. (2018). Temperature compensation in fluid density measurement using micro-electromechanical resonant sensor. Rev. Sci. Instrum..

[55] Hou G., Han Y., Xie B., Wei G., Li Z., Xiao H., Zhou T. (2019). Study on pretension strain loss of fixed point optical fiber in tunnel structure health monitoring. Rock Soil Mech..

[56] Zhou z., Chen T. (2021). A High Accuracy Monitoring Method of Multi object Dynamic Displacement Based on Computer Vision in Disturbance Environment. J. Vib. Eng..

[57] Ding Y., Zhou S., Dong J., Wang Z., Zheng Z. (2019). Application of Artificial Intelligence Method in Civil Engineering Monitoring. Mater. Rep..

[58] Li B., Wang G., Yuan J. (2021). Nonlinear deformation prediction model based on correlated monitoring data. J. Vib. Shock.

[59] Liu Q., Yang C.-Y., Lin L. (2021). Deformation Prediction of a Deep Foundation Pit Based on the Combination Model of Wavelet Transform and Gray BP Neural Network. Math. Probl. Eng..

[60] Hoseinian F.S., Abdollahzadeh A., Rezai B. (2018). Semi-autogenous mill power prediction by a hybrid neural genetic algorithm. J. Cent. South Univ..

[61] Yaman M.A., Abd Elaty M., Taman M. (2017). Predicting the ingredients of self compacting concrete using artificial neural network. Alex. Eng. J..

[62] Qian M., Li Y., He R., Zeng H. (2021). Measurement Method of Railway Network Scale Based on MI Ranger NARX Fusion Model. J. China Railw. Soc..

[63] Ma Q., Liu S., Fan X., Chai C., Wang Y., Yang K. (2020). A time series prediction model of foundation pit deformation based on empirical wavelet transform and NARX network. Mathematics.

[64] Cui A., Xu H., Jia P. (2011). An Elman neural network-based model for predicting anti-germ performances and ingredient levels with limited experimental data. Expert Syst. Appl..

[65] Guo J., Chen J., Hu Y. (2020). Time series prediction analysis of subway station foundation pit deformation based on wavelet intelligent model. Rock Soil Mech..

[66] Zhang Y., Zhang G., Li J., Cao Y., Hao B. (2021). Research on Deep Foundation Pit Online Monitoring Platform Based on Internet of Things and WebGIS. Chin. J. Undergr. Space Eng..

